# Application of Electrically Conducting Nanocomposite Material Polythiophene@NiO/Frt/GOx as Anode for Enzymatic Biofuel Cells

**DOI:** 10.3390/ma13081823

**Published:** 2020-04-12

**Authors:** Inamuddin, Khalid A. Alamry

**Affiliations:** 1 Chemistry Department, Faculty of Science, King Abdulaziz University, Jeddah 21589, Saudi Arabia; k_alamry@yahoo.com; 2 Advanced Functional Materials Laboratory, Department of Applied Chemistry, Faculty of Engineering and Technology, Aligarh Muslim University, Aligarh 202 002, India

**Keywords:** biofuel cells, enzyme, glucose oxidase, biofuel, anode

## Abstract

In this work, nano-inspired nickel oxide nanoparticles (NiO) and polythiophene (Pth) modified bioanode was prepared for biofuel cell applications. The chemically prepared nickel oxide nanoparticles and its composite with polythiophene were characterized for elemental composition and microscopic characterization while using scanning electron microscopy. The electrochemical characterizations of polythiophene@NiO composite, biocompatible mediator ferritin (Frt) and glucose oxidase (GOx) catalyst modified glassy carbon (GC) electrode were carried out using cyclic voltammetry (CV), linear sweep voltammetry (LSV), and charge-discharge studies. The current density of Pth@NiO/Frt/GOx bioanode was found to be 5.4 mA/cm^2^. The bioanode exhibited a good bio-electrocatalytic activity towards the oxidation of the glucose. The experimental studies of the bioanode are justifying its employment in biofuel cells. This will cater a platform for the generation of sustainable energy for low temperature devices.

## 1. Introduction

The advancement of technologies in the era of energy crisis are currently emphasising on the production of clean and green energy using efficient, sustainable and renewable resources [1]. This curiosity in research field leads to the development of enzymatic biofuel cells (EBFCs) [2]. The enzymatic biofuel cell generates energy from the biofuels e.g., glucose, fructose, alcohols, etc. by using the biocatalysts e.g., glucose oxidase, glucose dehydrogenase, etc. [3,4,5]. The advantages of EBFCs over the conventional fuel cells are less expensive, eco-friendly, and their favorable operational conditions, such as an ambient temperature and pressure [6,7,8,9,10], which make the EBFCs suitable for the small-scale power generation devices for in-vivo applications, like in miniaturized sensors, artificial organs, and implantable biomedical devices (e.g., cardiac pacemakers, insulin pumps) [11]. Glucose-based EBFC seems to be a promising candidate for implantable applications, due to the availability of glucose and oxygen in physiological fluids that make the possibility for the generation of electrical power inside the living system. The glucose used as a fuel oxidizes at the anode via redox enzyme, whereas the reduction of oxygen takes place at the cathode, to carry out redox reaction [12]. To date, various redox enzymes have been exploited, such as glucose dehydrogenase [13], fructose dehydrogenase [4], cellobiose dehydrogenase [14], and glucose oxidase (GOx) [3], as biocatalysts for the anode modification. Generally, the selectivity of enzyme depends on the substrate (fuel) used. In view of this, GOx has been exploited in glucose-based EBFCs. Additionally, the specificity of GOx (enzyme) towards glucose (substrate) leads to the development of a membrane-free system, which further boosts up the potential use of EBFCs in implantable devices due to the miniaturization of the system.

Despite all of these attributes, EBFCs are still facing some real-world challenges, such as the low magnitude of power output and less stability over time. These problems directly link to the poor enzyme sticking on to the electrode surface and delay in electrons transfer from the enzyme active site to the electrode surface, which in turn produces inadequate power to run various devices. The resistance in electrons transfer occurs because the active centre of GOx is in-depth of protein shell [15,16]. To overcome this issue, ferritin (Frt) has been utilized as a mediator in glucose-based EBFCs. Frt is an active redox protein that is biocompatible and eco-friendly, and one of the foremost benefits of using ferritin is that it is working at a redox potential near GOx. Hence, the aforementioned attributes are favorable in the application of Frt as mediator [17,18].

In recent times, the integration of nano-dimension materials and metallic nanoparticles in the design of electrode materials has been explored for EBFCs application [19]. For this purpose, nanomaterials have generated exceptional attention, owing to their high surface to volume ratio for enzyme loading, inspiring electronic properties and catalytic activity [20]. For instance, conducting polymers have been continuously explored as a new way for improving the performance of EBFCs [21]. The outstanding electronic properties, electrochemical stability, and biocompatibility make the conducting polymers ideal candidates for electrode catalyst support. The combinations of conducting polymers and metal nanoparticles (MNPs) have been endlessly published because of these attributes [22,23]. The synergistic effects of conducting polymers and metal nanoparticles improve the overall performance of the desired results [24]. The outstanding properties of MNPs, such as high conductivity, large surface area, and high catalytic activity, make them feasible in utilizing them as electrode materials. So far, titanium oxide (TiO_2_) [25], magnetite (Fe_3_O_4_) [26], silver (Ag) [27], and gold (Au) [28] nanoparticles in combination with conducting polymers have been successfully used. Nowadays, nickel oxide nanoparticles (NPs) have been utilized in various research areas, for example, gas sensors [29], catalysis [30], thermoelectric, pharmaceutical, cosmetic industries [31] solar [32], and semiconductors [33], due their attractive physicochemical properties, such as high mechanical and thermal stabilities, radiation hardness, and easy processing. Herein, a binary nanocomposite by exploiting the properties of NiO NPs and polythiophene is prepared. The synthesized nanocomposite was employed as support material for the mediator and enzyme.

## 2. Materials and Methods

Nickel chloride hexahydrate (NiCl_2_·6H_2_O), ethanol (C_2_H_5_OH), ferric chloride (FeCl_3_), ferritin (10 mg/mL in 0.15 M sodium chloride from horse spleen), KOH, and glutaraldehyde (Glu) were purchased from Sigma–Aldrich, (India). Phosphate buffer saline (PBS of pH 7.0 and 5.0), cetyl trimethyl ammonium bromide (CTAB), glucose oxidase (GOx) having activity from 100,000 to 150,000 units g^−1^ of protein supplied by Central Drug House (CDH), (India) were used. Thiophene monomer was obtained from Merck, India. Double distilled water (DDW) was utilized throughout the investigations. The electrochemical studies were carried out in a three electrode system while using modified glassy carbon electrode (GC) as working, a Pt wire as a counter, and Ag/AgCl (3 M KCl) as a reference electrode, coupled with the potentiostat/galvanostat (PGSTAT 302 Autolab, Switzerland).

### 2.1. Synthesis of NiO

The NiO particles were synthesized using 20 mL of 0.1 M NiCl_2_·6H_2_O in absolute ethanol, which were further added to a 6.73 mL of 5.0 M hydrazine monohydrate solution. The pH was maintained from 8.0 to 12 with the help of KOH. The reaction was left at room temperature on agitation for 2 h. The obtained product was thoroughly washed with double distilled water, followed by washing with acetone for the elimination of residues. Deep green [Ni(OH)_2_·0.5H_2_O] particles were obtained, which were kept in the vacuum oven for drying. Through thermal decomposition, the Ni(OH)_2_·0.5H_2_O particles were converted into the NiO particles at 650 °C [34,35].

### 2.2. Synthesis of Polythiophene (Pth)

The Pth matrix was synthesized by the chemical polymerization method. A 1.0 g of thiophene monomer and 0.004 mol of CTAB were dissolved in 50 mL of double distilled water with continuous stirring for 20 min. A 50 mL of 0.06 M FeCl_3_ aqueous solution utilized as oxidant was added drop-by-drop to the mixture of thiophene and surfactant under stirring. The beginning of the polymerization was checked by color change to dark brown after 24 h of stirring at 30 °C. The dark-brown precipitate of Pth was washed with double distilled water (DDW) and methanol until colorless filtrate was found. The Pth precipitate was dried in hot air oven for 8 h at 45 °C.

### 2.3. Preparation of Composite Dispersion

The above-prepared NiO particles and Pth powder (100 mg each) were dissolved in 2 mL double distilled water (DDW). The mixture was sonicated in a vial for 1 h to obtain the dispersion of Pth@NiO. The performance of dispersion was evaluated by making the use of a UV–vis spectrophotometer and an absorption spectrum between 300–700 nm wavelengths was recorded.

### 2.4. Preparation of the Electrode

**Scheme 1:**
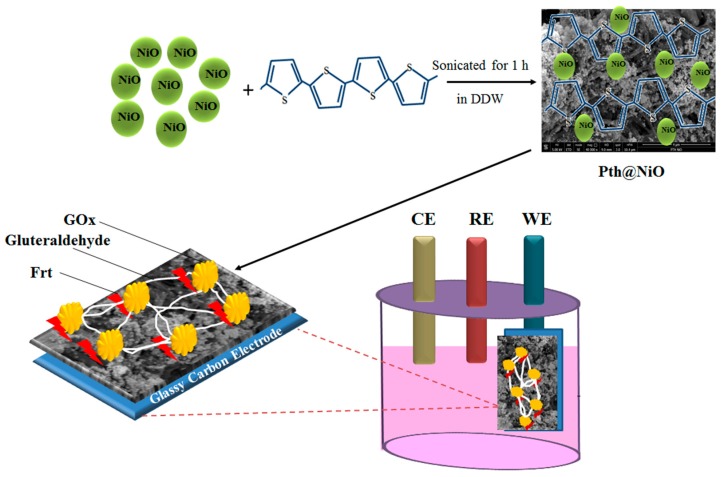
The schematic representation for the preparation of bioanode and electrode arrangements in electrochemical cell.

The glassy carbon electrode (GCE) was cleaned on a velvet pad with the help of alumina powder (0.05 µm). After polishing, the electrode was kept in ultrasonicator bath for 30 min., followed by the washing from distilled water, and finally left to dry at room temperature. The GC electrode was modified by 6.0 µL dropwise coating of the composite dispersion and left to dry at room temperature for 6 h. Further, 3 µL of Frt was drop casted on the Pth@NiO modified electrode, which was kept for drying at room temperature. A 10 mg/mL GOx was prepared in PBS of pH 5.0 to sustain the enzyme activity during the immobilization technique. The GOx solution (10 µL) was coated dropwise on Pth@NiO/Frt modified GC electrode and then left to efficiently immobilize on the composite for 2 h. Lastly, a 1.0 µL of 3% glutaraldehyde aqueous solution was drop casted for the purpose of crosslinking among the materials applied on the GC electrode and kept for drying. The GC electrode that was modified with Pth@NiO/Frt/GOx was dipped in DDW for 60 s to remove any unbound GOx. The electrode was kept in the refrigerator at 4 °C prior to use. Scheme 1 provides the schematic representation for the preparation of bianode.

## 3. Results and Discussion

### 3.1. SEM and EDX Analyses

**Figure 1:**
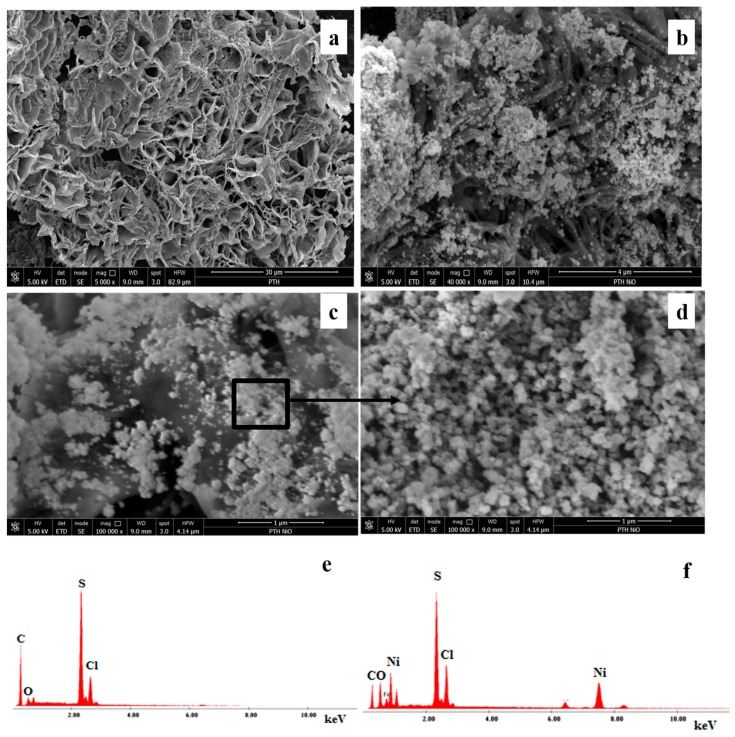
Scanning electron microscopy (SEM) and energy-dispersive X-ray spectroscopy (EDX) pictures of (**a**) Pth, (**b**–**d**) Pth@NiO, and (**e**) Pth, (**f**) Pth@NiO, respectively.

The scanning electron microscopy (SEM) pictures clearly show the involvement of NiO in Pth matrix, which was also analyzed by energy-dispersive X-ray spectroscopy (EDX). EDX analysis of Pth and Pth@NiO displayed that NiO particles are evenly dispersed in the polymeric matrix that can be seen in the SEM images in Figure 1a,b. Figure 1a displays the morphology of Pth matrix and, on the other hand, Figure 1b confirms the incorporation of NiO particles in Pth matrix, which is further assured by SEM micrograph of Figure 1c,d at higher magnification. EDX surface mapping supports the confirmation of SEM analysis of Pth and Pth@NiO by indicating the presence of elemental composition. Figure 1f exhibits the presence of inorganic NiO particles in polymer matrix, in addition to C and S [36,37].

### 3.2. Electrochemical Analyses

**Figure 2:**
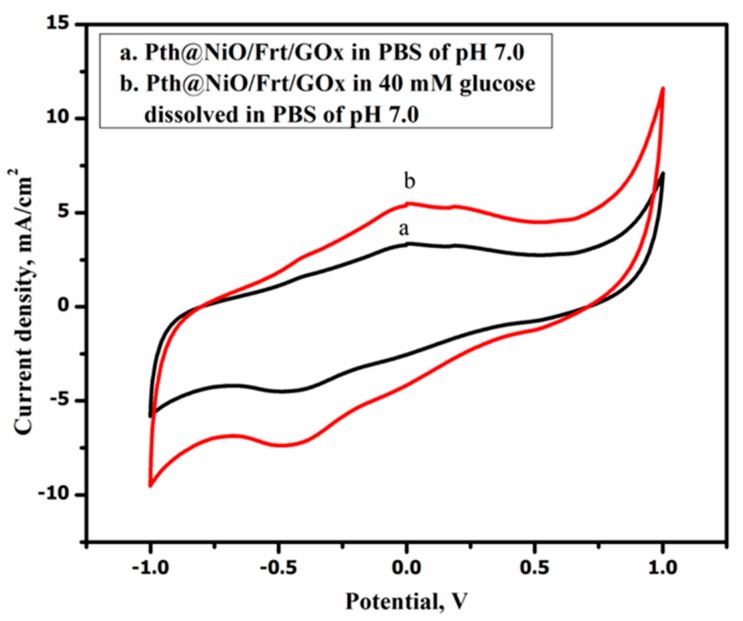
Cylic voltammograms of Pth@NiO/Frt/GOx modified glassy carbon (GC) electrode, a: in phosphate buffer saline (PBS) of pH 7.0, b: in 40 mM glucose dissolved in PBS of pH 7.0.

The cyclic voltammetry investigation of the Pth@NiO/Frt/GOx bioelectrode was carried out to analyze the electrical communication of the generated electrons via mediated electron transfer from the enzyme redox active site to the glassy carbon modified electrode. The cyclic voltammetry curves depicted in Figure 2 displayed the glassy carbon modified electrode with Pth@NiO/Frt/GOx showing a significant electrocatalytic redox activity. This might be due to the Frt resulting in a better dispersion of the composite, causing an enhancement in the bioelectrocatalytic reaction of the modified glassy carbon electrode [38]. The cyclic voltammetry studies of Pth@NiO/Frt/GOx GC modified electrode at a scan rate of 100 mV/s in PBS of pH 7.0 provides a current of 3.2 mA/cm^2^, whereas repeating the same experiment in presence of 40 mM glucose that was pre-optimized, the current has increased to 5.4 mA/cm^2^. This clearly indicates that the combination of Frt and GOx provided the required redox reaction, causing electron transfer towards the Pth@NiO modified glassy carbon electrode. The outstanding electrical connection came into existence due to the synergistic effects of the composite that allows for the easier shuttling of electrons from deeply buried redox active sites of GOx to the surface of electrode [3,39]. The redox property of the composite emerged due to the incorporation of NiO significantly enhances the catalytic activity towards glucose oxidation reaction. Nevertheless, Ni- based material has poor electrical conductivity, hence increasing the charge transfer resistance of the electrode. For that reason, Pth are used as a support to enhance its electrical property along with improved surface area [40]. The composite has offered the required mechanical and electrical properties due to the incorporation of NiO on Pth, which contributes towards exceptional electrocatalytic activity for glucose substrate. 

**Figure 3:**
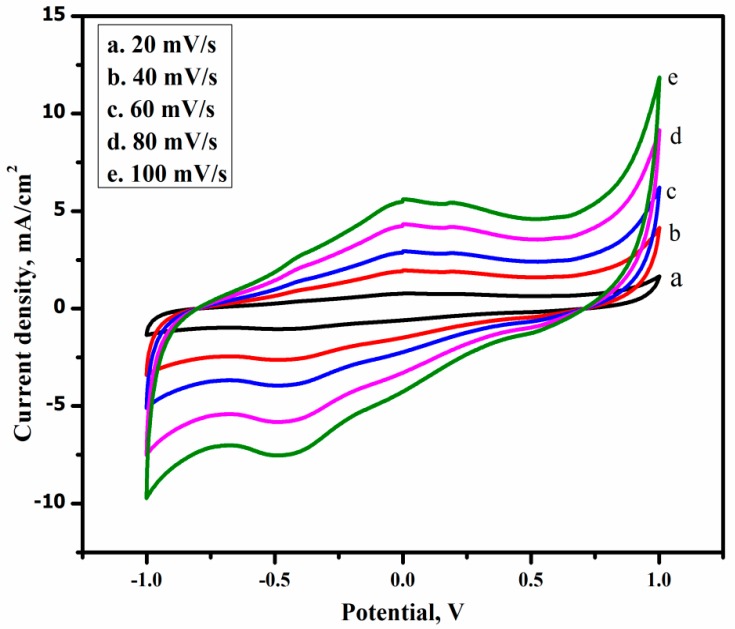
Cyclic voltammograms of Pth@NiO/Frt/GOx modified glassy carbon electrode in 40 mM glucose solution PBS of pH 7.0 at scan rate a: 20, b: 40, c: 60, d: 80, and e 100 mV/s.

**Figure 4:**
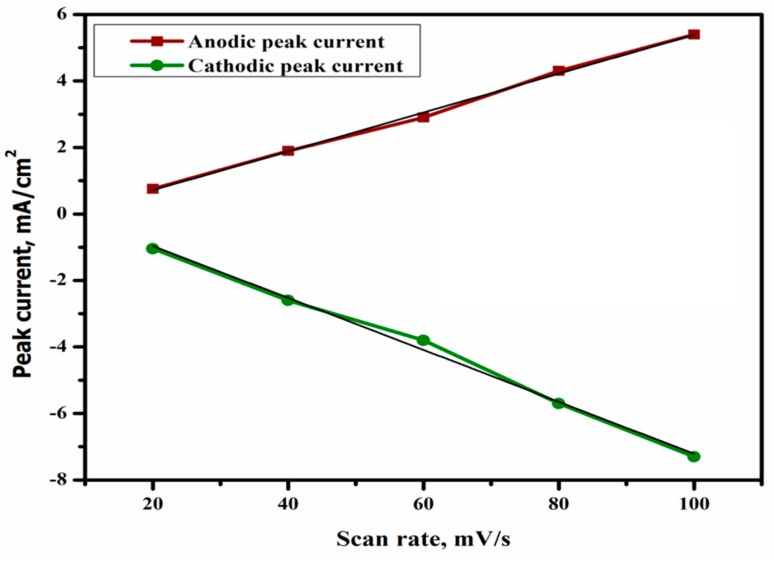
Plot of peak current vs. scan rate (20–100 mV/s).

The electrochemical behavior of the glassy carbon electrode modified Pth@NiO/Frt/GOx was analyzed for the several scan rates (20, 40, 60, 80, and 100 mV/s) in 40 mM glucose dissolved in PBS of pH 7.0, as shown in Figure 3. As the scan rate increased, the peak current is also increased. The cyclic voltammograms showed the linear relationship between the oxidation/reduction peaks current and scan rate. Figure 4 showed that the oxidation/reduction peak currents are in direct relation with the scan rate, because the increasing concentration gradient resulted in a diffusion-controlled reaction. Thus, it can be concluded that the scan rate is one of the dominant aspects that governs the bioelectrocatalytic effectiveness of the fabricated bioelectrode. Furthermore, cyclic voltammetry hysteresis of the Pth@NiO/Frt/GOx biocomposite electrode was still maintained on elevated scan rate and it signifies the application of the composite as an efficient electrode material for biofuel cells [9,41]. This might be likely because of the integration of NiO particles in Pth matrix, offering a high surface area and enhancement in the electrochemical activity and electrical conductivity of the prepared Pth@NiO/Frt/GOx bioanode.

The rate constant, *Ks* (2.0 s^−1^) for electron transfer from the GOx enzyme onto the Pth@NiO modified bioelectrode was computed from the well-known Laviron equation, as given below [42].

(1) logks=αlog(1−α)+(1−α)logα−log(RT/nFv)−α(1−α)(nFΔEp/2.3RT)

where *α* = 0.5, the constants *R*, *T*, and *F* express their usual meanings (*R* = 8.314 JK^−1^ mol^−1^, *T* = 298 K, *F* = 96485 C mol^−1^), no. of electron transfer, *n* = 2, scan rate, *v* (100 mV/s), Δ*Ep* = *Ep*a − *Ep*c.

The surface concentration, I^*^ of the adsorbed electroactive Pth-NiO/Frt/GOx modified bioanode was estimated by using Brown–Anson model by Equation (1), as shown below [43].

I_p_ = *n*^2^*F*^2^I^*^A*V*/4*RT* (2)

where *n* represents the number of electrons migrated (in the present case *n* = 2), *F* meant for the Faraday constant (96,485 C mol^−1^), I^*^ is the surface concentration of the redox species to be evaluated on the surface of Pth@NiO/Frt/GOx biocomposite electrode (mol/cm^2^), A symbolizes the area of glassy carbon electrode (0.07 cm^2^), *v* is the scan rate (100 mV/s), and *T* and *R* have their universal meaning. The surface concentration of the GOx that was confined on Pth@NiO/Frt bioanode was estimated to be 2.053 × 10^−7^ mol/cm^2^.

**Figure 5:**
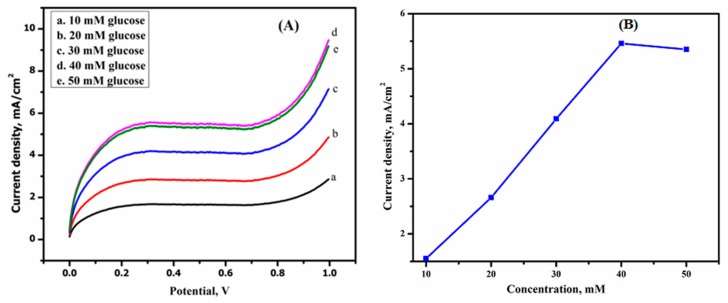
(**A**) Linear sweep voltammetry (LSV) curves of Pth@NiO/Frt/GOx modified glassy carbon electrode in PBS of pH 7.0 containing different concentrations of glucose *viz*, a. 10 mM, b, 20 mM, c. 30 mM, d. 40 mM, and e. 50 mM at room temperature with a potential scan rate of 100 mV/s, (**B**) the calibration curve corresponding to the electrocatalytic current against variable concentration of glucose.

The current response of the Pth@NiO/Frt/GOx modified glassy carbon electrode was studied with respect to various glucose concentrations that ranged from 10–50 mM in PBS of pH 7.0, as shown in Figure 5A. The increase in current density was observed with the successive addition of glucose concentration up to 40 mM, beyond that the redox current acquired saturation because the bioelectrocatalytic redox reaction was slowed down by the higher glucose concentration. This saturated current might be due to the saturation kinetics of the redox reaction, which is offering steady-state equilibrium in the redox current and remains stable, even after the further increment in the glucose concentration [3,39,44]. Figure 5B shows a typical calibration curve for the oxidation peak current vs. the glucose concentration. This curve exhibits linear enhancement in the current density with the increase in glucose concentration and subsequently the bioanode reached a saturation current of 5.4 ± 0.5 mA/cm^2^ for the biocatlytic oxidation of glucose at the 40 mM glucose concentration. These results undoubtedly point toward the successful biocatalytic activity of the biocomposite electrode in the presence of glucose. Therefore, the fabricated Pth@NiO/Frt/GOx bioelectrode can also be utilized in the field of glucose biosensors.

**Figure 6:**
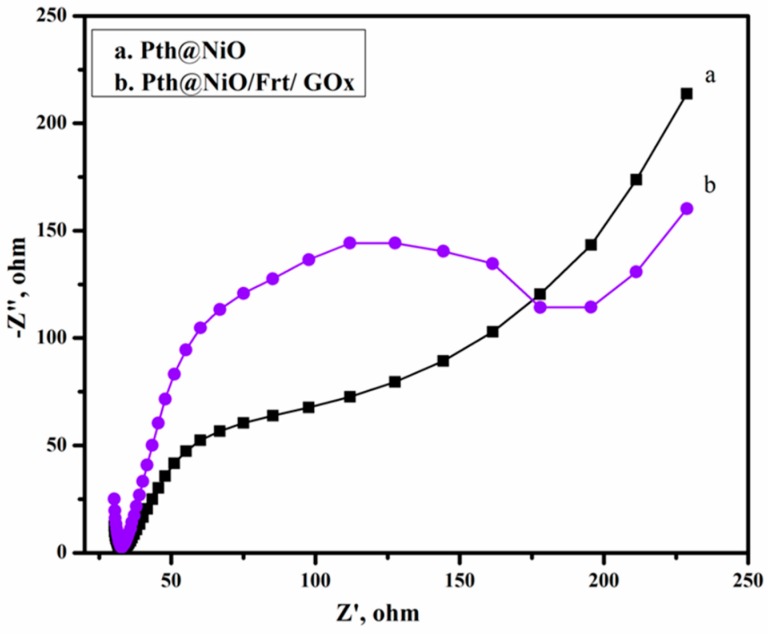
Electrochemical impedance spectroscopy (EIS) curves for a. Pth@NiO, b. Pth@NiO/Frt/GOx modified GC electrode in 0.1 M K_4_Fe(CN)_6._

Electrochemical impedance spectroscopy (EIS) was carried out to study the behavior of the Pth@NiO and Pth@NiO/Frt/GOx at the electrode-electrolyte interface. The imaginary (−Z″) and real (Z′) component of the EIS spectra showed the Nyquist plot for Pth@NiO and Pth@NiO/Frt/GOx modified GC electrodes in 0.1 M KCl solution having 5.0 mM K_4_Fe(CN)_6_. In high to medium frequency region, a semicircle is obtained, which indicates the resistance to charge transfer. Whereas the straight line at low frequency signifies the reaction is diffusion controlled. Pth@NiO modified electrode generated a circle with small diameter that clearly indicates low resistance to charge transfer (R_ct_ = 80 Ω), as shown in Figure 6. The lower value of R_ct_ of the Pth@NiO is a sign of good electrical conductivity and contact of the Pth@NiO with glassy carbon electrode. Further, Pth@NiO/Frt/GOx showed R_ct_ = 145 Ω, which is relatively bigger semi-circle and signifies for its larger value of resistance to charge transfer, which confirms the suitable immobilization of the enzyme and mediator on the electrode surface [45].

**Figure 7:**
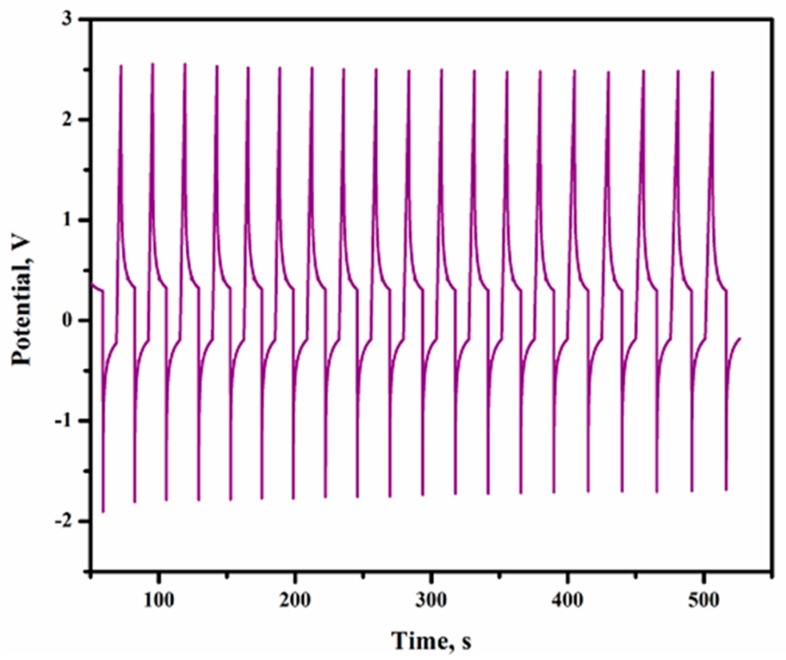
Galvanostatic charge-discharge curve for Pth@NiO/Frt/GOx modified GC electrode at 5.4 ± 0.5 mA/cm^2^.

Figure 7 displays the galvanostatic charge-discharge curves of Pth@NiO/Frt/GOx modified GC bioelectrode at 5.4 ± 0.5 mA/cm^2^. All of the curves of charge-discharge are almost alike in symmetry during the operation cycle. These curves clearly suggested significant pseudocapacitive properties of the composite Pth@NiO. The reversibility of charge-discharge for bioelectrode confirms the synergic involvement of the pseudocapacitance and capacitance of the hybrid material along with its high conductivity.

## 4. Conclusions

The prepared biocomposite electrode exhibited exceptional electrochemical behavior due to the presence of Pth@NiO conductive support. It has been found that the fruitful oxidation of glucose to gluconolactone with the generation of appreciable current density was achieved while using Pth@NiO/Frt/GOx GC modified bioanode. The current density that was obtained with this material was found to be 5.4 mA/cm^2^. The Pth@NiO/Frt/GOx bioanode exhibited the good bio-electrocatalytic activity towards the oxidation of the glucose substrate supported by Frt mediator, which played an essential role for increasing the electron transfer rate. The bioelectrode also showed the long- lasting stability due to the incorporation of Pth@NiO that imparts the suitable environment for the proper functioning of enzyme. Thus, the Pth@NiO/Frt/GOx bioelectrode showed a promising approach for being a better option to employ in implantable biomedical devices.
